# Supplementary material for: Playing in ‘The Backyard’: Environmental Features and Conditions of a Natural Playspace Which Support Diverse Outdoor Play Activities among Younger Children
Source: Int J Environ Res Public Health. 2022 Oct 3;19(19):12661. doi: 10.3390/ijerph191912661 (PMC9565111; doi:10.3390/ijerph191912661)
Supplement: Supplementary file 1 [file ijerph-19-12661-s001.zip › ijerph-1822840-supplementary.pdf]

Supplementary Materials

Figure S1. Behavior Map of all Bio, Expressive, Restorative & Digital Play Activities.

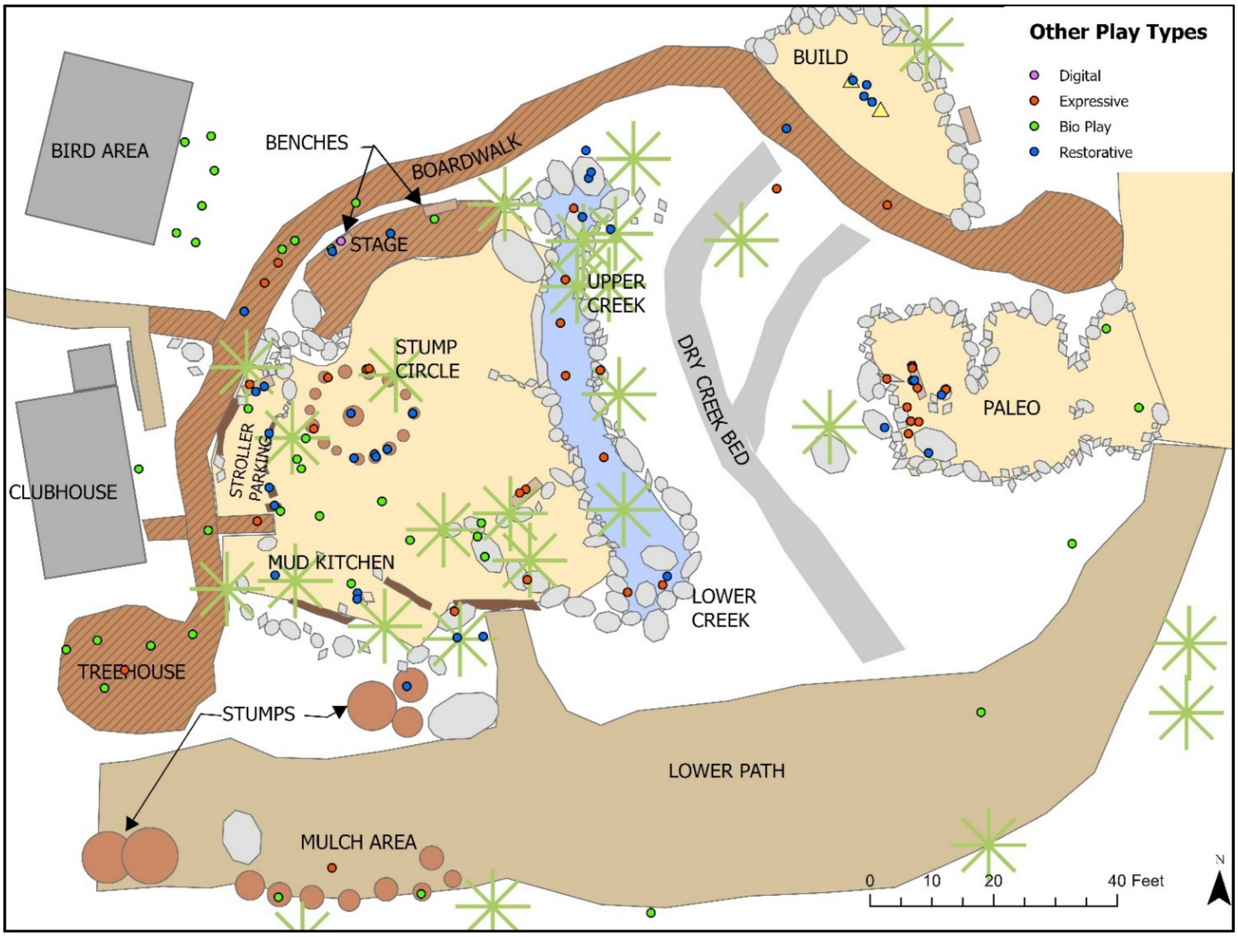

Table S1. Two-Way Associations between Outdoor Play Types and Select Demographic, Behavioral and Environmental Variables

	OUTDOOR PLAY TYPES									
	**Physical**	**Exploratory**	**Imaginative**	**Play with Rules**	**Bio**	**Expressive**	**Restorative**	**Digital**	**Non-play**	**Total**
**GENDER**										
Male (n)	280	249	35	38	20	10	14	1	54	701
% across all OPTs	*39.9*	*35.5*	*5.0*	*5.4*	*2.9*	*1.4*	*2.0*	*0.1*	*7.7*	
% within OPT	*64.5*	*59.9*	*52.2*	*80.9*	*51.3*	*28.6*	*38.9*	*100.0*	*44.3*	
Female (n)	154	167	32	9	19	25	22	0	68	496
% across all OPTs	*31.0*	*33.7*	*6.5*	*1.8*	*3.8*	*5.0*	*4.4*	*0.0*	*13.7*	
% within OPT	*35.5*	*40.1*	*47.8*	*19.2*	*48.7*	*71.4*	*61.1*	*0.0*	*55.7*	
**AGE**										
3-8 years old (n)	368	364	67	47	38	33	31	1	104	1053
% across all OPTs	*34.9*	*34.6*	*6.4*	*4.5*	*3.6*	*3.1*	*2.9*	*0.1*	*9.9*	
% within OPT	*84.8*	*87.5*	*100.0*	*100.0*	*97.4*	*94.3*	*86.1*	*100.0*	*85.3*	
0-2 years old (n)	66	52	0	0	1	2	5	0	18	144
% across all OPTs	*45.8*	*36.1*	*0.0*	*0.0*	*0.7*	*1.4*	*3.5*	*0.0*	*12.5*	
% within OPT	*15.2*	*12.5*	*0.0*	*0.0*	*2.6*	*5.7*	*13.9*	*0.0*	*14.8*	
**CARS CONDENSED**										
Stationary (n)	93	174	29	7	31	24	30	1	62	451
% across all OPTs	*20.6*	*38.6*	*6.4*	*1.6*	*6.9*	*5.3*	*6.7*	*0.2*	*13.7*	
% within OPT	*21.4*	*41.8*	*43.3*	*14.9*	*79.5*	*68.6*	*83.3*	*100.0*	*50.8*	
Slow (n)	158	162	24	17	6	3	5	0	25	400
% across all OPTs	*39.5*	*40.5*	*6.0*	*4.3*	*1.5*	*0.8*	*1.3*	*0.0*	*6.3*	
% within OPT	*36.4*	*38.9*	*35.8*	*36.2*	*15.4*	*8.6*	*13.9*	*0.0*	*20.5*	
Moderate-Vigorous (n)	183	80	14	23	2	8	1	0	35	346
% across all OPTs	*52.9*	*23.1*	*4.0*	*6.6*	*0.6*	*2.3*	*0.3*	*0.0*	*10.1*	
% within OPT	*42.2*	*19.2*	*20.9*	*48.9*	*5.1*	*22.9*	*2.8*	*0.0*	*28.7*	
**RISK CONDENSED**										
No/Low Risk (n)	117	216	47	8	35	25	27	1	103	579
% across all OPTs	*20.2*	*37.3*	*8.1*	*1.4*	*6.0*	*4.3*	*4.7*	*0.2*	*17.8*	
% within OPT	*27.0*	*51.9*	*70.2*	*17.0*	*89.7*	*71.4*	*75.0*	*100.0*	*84.4*	
Positive Risk (n)	312	199	20	39	4	10	9	0	19	612
% across all OPTs	*51.0*	*32.5*	*3.3*	*6.4*	*0.7*	*1.6*	*1.5*	*0.0*	*3.1*	
% within OPT	*71.9*	*47.8*	*29.9*	*83.0*	*10.3*	*28.6*	*25.0*	*0.0*	*15.6*	
Negative Risk (n)	5	1	0	0	0	0	0	0	0	6
% across all OPTs	*83.3*	*16.7*	*0.0*	*0.0*	*0.0*	*0.0*	*0.0*	*0.0*	*0.0*	

% within OPT	*1.2*	*0.2*	*0.0*	*0.0*	*0.0*	*0.0*	*0.0*	*0.0*	*0.0*	

	**OUTDOOR PLAY TYPES**									
	**Physical**	**Exploratory**	**Imaginative**	**Play with Rules**	**Bio**	**Expressive**	**Restorative**	**Digital**	**Non-play**	**Total**
**INVOLVED LOOSE PARTS**										
Yes (n)	323	374	59	39	27	13	19	1	48	903
% across all OPTs	*35.8*	*41.4*	*6.5*	*4.3*	*3.0*	*1.4*	*2.1*	*0.1*	*5.3*	
% within OPT	*74.4*	*89.9*	*88.1*	*83.0*	*69.2*	*37.1*	*52.8*	*100.0*	*39.3*	
No (n)	111	42	8	8	12	22	17	0	74	294
% across all OPTs	*37.8*	*14.3*	*2.7*	*2.7*	*4.1*	*7.5*	*5.8*	*0.0*	*25.2*	
% within OPT	*25.6*	*10.1*	*11.9*	*17.0*	*30.8*	*62.9*	*47.2*	*0.0*	*60.7*	
**INVOLVED NATURAL LOOSE PARTS**										
Yes (n)	282	335	56	35	26	9	16	1	34	794
% across all OPTs	*35.5*	*42.2*	*7.1*	*4.4*	*3.3*	*1.1*	*2.0*	*0.1*	*4.3*	
% within OPT	*65.0*	*80.5*	*83.6*	*74.5*	*66.7*	*25.7*	*44.4*	*100.0*	*27.9*	
No (n)	152	81	11	12	13	26	20	0	88	403
% across all OPTs	*37.7*	*20.1*	*2.7*	*3.0*	*3.2*	*6.5*	*5.0*	*0.0*	*21.8*	
% within OPT	*35.0*	*19.5*	*16.4*	*25.5*	*33.3*	*74.3*	*55.6*	*0.0*	*72.1*	
**INVOLVED MANUFACTURED LOOSE PARTS**										
Yes (n)	210	229	49	37	4	6	6	1	22	564
% across all OPTs	*37.2*	*40.6*	*8.7*	*6.6*	*0.7*	*1.1*	*1.1*	*0.2*	*3.9*	
% within OPT	*48.4*	*55.1*	*73.1*	*78.7*	*10.3*	*17.1*	*16.7*	*100.0*	*18.0*	
No (n)	224	187	18	10	35	29	30	0	100	633
% across all OPTs	*35.4*	*29.5*	*2.8*	*1.6*	*5.5*	*4.6*	*4.7*	*0.0*	*15.8*	
% within OPT	*51.6*	*45.0*	*26.9*	*21.3*	*89.7*	*82.9*	*83.3*	*0.0*	*82.0*	
**TOPOGRAPHY**										
No/Low Slope (n)	174	222	45	7	32	19	23	1	85	608
% across all OPTs	*28.6*	*36.5*	*7.4*	*1.2*	*5.3*	*3.1*	*3.8*	*0.2*	*14.0*	
% within OPT	*40.1*	*53.4*	*67.2*	*14.9*	*82.1*	*54.3*	*63.9*	*100.0*	*69.7*	
Moderate Slope (n)	20	7	2	2	3	2	4	0	16	56
% across all OPTs	*35.7*	*12.5*	*3.6*	*3.6*	*5.4*	*3.6*	*7.1*	*0.0*	*28.6*	
% within OPT	*4.6*	*1.7*	*3.0*	*4.3*	*7.7*	*5.7*	*11.1*	*0.0*	*13.1*	
Steep Slope (n)	8	6	1	0	1	1	0	0	6	23
% across all OPTs	*34.8*	*26.1*	*4.3*	*0.0*	*4.3*	*4.3*	*0.0*	*0.0*	*26.1*	

% within OPT	*1.8*	*1.4*	*1.5*	*0.0*	*2.6*	*2.9*	*0.0*	*0.0*	*4.9*	
Uneven Surface (n)	232	181	19	38	3	13	9	0	15	510
% across all OPTs	*45.5*	*35.5*	*3.7*	*7.5*	*0.6*	*2.5*	*1.8*	*0.0*	*2.9*	
% within OPT	*53.5*	*43.5*	*28.4*	*80.9*	*7.7*	*37.1*	*25.0*	*0.0*	*12.3*	
**Total**	**434**	**416**	**67**	**47**	**39**	**35**	**36**	**1**	**122**	**1197**

OPT = Outdoor Play Type

Table S2: Associations with Outdoor Play Types for Select Demographic & Behavioral Variables using Pearson Chi2 & Fisher's Exact Test

PHYSICAL PLAY	Gender		Age		CARS (condensed)			Risk (condensed)			*Totals*
	Female	*Male*	*0-2 yrs*	*3-8 yrs*	*Stationary*	*Slow*	*ModVig*	*No/Low*	*Positive*	*Negative*	
No Count	133	126	20	239	159	73	27	205	54	0	259
Expected Count	*107.3*	*151.7*	*32.1*	*226.9*	*94.2*	*86.3*	*78.5*	*120.3*	*136.8*	*1.9*	
Yes Count	154	280	66	368	93	158	183	117	312	5	434
Expected Count	*179.7*	*254.3*	*53.9*	*380.1*	*157.8*	*144.7*	*131.5*	*201.7*	*229.2*	*3.1*	
**Total**	**287**	**406**	**86**	**607**	**252**	**231**	**210**	**322**	**366**	**5**	**693**
Pearson chi2 (p)	16.8318	**0.000*****	8.3611	**0.004****	130.3137	**0.000*****		178.0828	**0.000*****		
Cramer's V	0.1558		-0.1098		0.4324			0.5069			
Fisher's exact		**0.000*****		**0.004****		**0.000*****			**0.000*****		

EXPLORATORY PLAY	Gender		Age		CARS (condensed)			Risk (condensed)			*Totals*
	Female	*Male*	*0-2 yrs*	*3-8 yrs*	*Stationary*	*Slow*	*ModVig*	*No/Low*	*Positive*	*Negative*	
No	120	157	34	243	78	69	130	106	167	4	277
Expected	*114.7*	*162.3*	*34.4*	*242.6*	*100.7*	*92.3*	*83.9*	*128.7*	*146.3*	*2*	
Yes	167	249	52	364	174	162	80	216	199	1	416
Expected	*172.3*	*243.7*	*51.6*	*364.4*	*151.3*	*138.7*	*126.1*	*193.3*	*219.7*	*3*	
**Total**	**287**	**406**	**86**	**607**	**252**	**231**	**210**	**322**	**366**	**5**	**693**
Pearson chi2 (p)	0.6918	0.406	0.0078	0.930	60.4703	**0.000*****		14.8944	**0.001****		
Cramer's V	0.0316		-0.0034		0.2954			0.1466			
Fisher's exact		0.431		1.000		**0.000*****			**0.000*****		

IMAGINATIVE PLAY	Gender		Age		CARS (condensed)			Risk (condensed)			*Totals*
	Female	*Male*	*0-2 yrs*	*3-8 yrs*	*Stationary*	*Slow*	*ModVig*	*No/Low*	*Positive*	*Negative*	
No	255	371	86	540	223	207	196	275	346	5	626
Expected	*259.3*	*366.7*	*77.7*	*548.3*	*227.6*	*208.7*	*189.7*	*290.9*	*330.6*	*4.5*	
Yes	32	35	0	67	29	24	14	47	20	0	67
Expected	*27.7*	*39.3*	*8.3*	*58.7*	*24.4*	*22.3*	*20.3*	*31.1*	*35.4*	*0.5*	
**Total**	**287**	**406**	**86**	**607**	**252**	**231**	**210**	**322**	**366**	`	**693**
Pearson chi2 (p)	1.2315	0.267	10.5086	**0.001****	3.2806	0.194		16.8951	**0.000*****		
Cramer's V	-0.0422		0.1231		0.0688			0.1561			
Fisher's exact		0.297		**0.000*****		0.183			**0.000*****		

PLAY W RULES	Gender		Age		CARS (condensed)			Risk (condensed)			*Totals*
	Female	*Male*	*0-2 yrs*	*3-8 yrs*	*Stationary*	*Slow*	*ModVig*	*No/Low*	*Positive*	*Negative*	
No	278	368	86	560	245	214	187	314	327	5	646
Expected	*267.5*	*378.5*	*80.2*	*565.8*	*234.9*	*215.3*	*195.8*	*300.2*	*341.2*	*4.7*	
Yes	9	38	0	47	7	17	23	8	39	0	47
Expected	*19.5*	*27.5*	*5.8*	*41.2*	*17.1*	*15.7*	*14.2*	*21.8*	*24.8*	*0.3*	
**Total**	**287**	**406**	**86**	**607**	**252**	**231**	**210**	**322**	**366**	**5**	**693**
Pearson chi2 (p)	10.3017	**0.001****	7.1435	**0.008****	12.2899	**0.002****		18.4574	**0.000*****		
Cramer's V	0.1219		0.1015		0.1332			0.1632			
Fisher's exact		**0.001****		**0.002****		**0.002****			**0.000*****		

**BIO PLAY**	**Gender**		**Age**		**CARS (condensed)**			**Risk (condensed)**			***Totals***
	Female	***Male***	***0-2 yrs***	***3-8 yrs***	***Stationary***	***Slow***	***ModVig***	***No/Low***	***Positive***	***Negative***	
No	268	386	85	569	221	225	208	287	362	5	654
Expected	*270.8*	*383.2*	*81.2*	*572.8*	*237.8*	*218*	*198.2*	*303.9*	*345.4*	*4.7*	
Yes	19	20	1	38	31	6	2	35	4	0	39
Expected	*16.2*	*22.8*	*4.8*	*34.2*	*14.2*	*13*	*11.8*	*18.1*	*20.6*	*0.3*	
**Total**	**287**	**406**	**86**	**607**	**252**	**231**	**210**	**322**	**366**	**5**	**693**
Pearson chi2 (p)	0.9086	0.340	3.6855	0.055+	33.771	**0.000*****		31.129	**0.000*****		
Cramer's V	-0.0362		0.0729		0.2208			0.2119			
Fisher's exact		0.403		0.075		**0.000*****			**0.000*****		

**EXPRESSIVE PLAY**	**Gender**		**Age**		**CARS (condensed)**			**Risk (condensed)**			***Totals***
	Female	***Male***	***0-2 yrs***	***3-8 yrs***	***Stationary***	***Slow***	***ModVig***	***No/Low***	***Positive***	***Negative***	
No	262	396	84	574	228	228	202	297	356	5	658
Expected	*272.5*	*385.5*	*81.7*	*576.3*	*239.3*	*219.3*	*199.4*	*305.7*	*347.5*	*4.7*	
Yes	25	10	2	33	24	3	8	25	10	0	35
Expected	*14.5*	*20.5*	*4.3*	*30.7*	*12.7*	*11.7*	*10.6*	*16.3*	*18.5*	*0.3*	
**Total**	**287**	**406**	**86**	**607**	**252**	**231**	**210**	**322**	**366**	**5**	**693**
Pearson chi2 (p)	13.6866	**0.000*****	1.5203	0.218	17.9705	**0.000*****		9.3118	**0.01***		
Cramer's V	-0.1405		0.0468		0.161			0.1159			
Fisher's exact		**0.000*****		0.296		**0.000*****			**0.008****		

**RESTORATIVE PLAY**	**Gender**		**Age**		**CARS (condensed)**			**Risk (condensed)**			***Totals***
	Female	***Male***	***0-2 yrs***	***3-8 yrs***	***Stationary***	***Slow***	***ModVig***	***No/Low***	***Positive***	***Negative***	
No	265	392	81	576	222	226	209	295	357	5	657
Expected	*272.1*	*384.9*	*81.5*	*575.5*	*238.9*	*219*	*199.1*	*305.3*	*347*	*4.7*	
Yes	22	14	5	31	30	5	1	27	9	0	36
Expected	*14.9*	*21.1*	*4.5*	*31.5*	*13.1*	*12*	*10.9*	*16.7*	*19*	*0.3*	
**Total**	**287**	**406**	**86**	**607**	**252**	**231**	**210**	**322**	**366**	**5**	**693**
Pearson chi2 (p)	6.0719	**0.014***	0.0764	0.782	36.8387	**0.000*****		12.4906	**0.002****		
Cramer's V	-0.0936		-0.0105		0.2306			0.1343			
Fisher's exact		**0.015***		0.796		**0.000*****			**0.002****		

**DIGITAL PLAY**	**Gender**		**Age**		**CARS (condensed)**			**Risk (condensed)**			***Totals***
	Female	***Male***	***0-2 yrs***	***3-8 yrs***	***Stationary***	***Slow***	***ModVig***	***No/Low***	***Positive***	***Negative***	
No	287	405	86	606	251	231	210	321	366	5	692
Expected	*286.6*	*405.4*	*85.9*	*606.1*	*251.6*	*230.7*	*209.7*	*321.5*	*365.5*	*5*	
Yes	0	1	0	1	1	0	0	1	0	0	1
Expected	*0.4*	*0.6*	*0.1*	*0.9*	*0.4*	*0.3*	*0.3*	*0.5*	*0.5*	*0*	
**Total**	**287**	**406**	**86**	**609**	**252**	**231**	**210**	**322**	**366**	**5**	**693**
Pearson chi2 (p)	0.7079	0.400	0.1419	0.706	1.7525	0.416		1.1538	0.562		
Cramer's V	0.032		0.0143		0.0503			0.0408			
Fisher's exact		1.000		1.000		1.000			0.472		

NON PLAY	Gender		Age		CARS (condensed)			Risk (condensed)			Totals
	Female	*Male*	*0-2 yrs*	*3-8 yrs*	*Stationary*	*Slow*	*ModVig*	*No/Low*	*Positive*	*Negative*	
No	219	352	68	503	190	206	175	219	347	5	571
Expected	*236.5*	*334.5*	*70.9*	*500.1*	*207.6*	*190.3*	*173*	*265.3*	*301.6*	*4.1*	
Yes	68	54	18	104	62	25	35	103	19	0	122
Expected	*50.5*	*71.5*	*15.1*	*106.9*	*44.4*	*40.7*	*37*	*56.7*	*64.4*	*0.9*	
**Total**	**287**	**406**	**86**	**607**	**252**	**231**	**210**	**322**	**366**	**5**	**693**
Pearson chi2 (p)	12.5204	**0.000*** **	0.7486	0.387	15.9616	**0.000*** **		85.8709	**0.000*** **		
Cramer's V	-0.1344		-0.0329		0.1518			0.352			
Fisher's exact		**0.001** **		0.368		**0.000*** **			**0.000*** **		

+ p ~ 0.05 ** p< 0.01

* p < 0.05 ***p<0.001
